# Fully automated CT-based quantitative body composition analysis for predicting survival in patients with HCC undergoing TACE: a dual-cohort study

**DOI:** 10.1038/s41598-026-56245-7

**Published:** 2026-06-16

**Authors:** Krzysztof Bartnik, Belgutei Tsevegmed, Kamil Książek, Zuzanna Wojtczak, Przemysław Biecek

**Affiliations:** 1 Second Department of Radiology, Medical University of Warsaw, Banacha 1a St, Warsaw, 02-097 Poland; 2 Centre for Credible Artificial Intelligence, Warsaw University of Technology, Warsaw, Poland

**Keywords:** Biomarkers, Cancer, Medical research, Oncology, Risk factors

## Abstract

Transarterial chemoembolization (TACE) is a standard treatment for patients with unresectable hepatocellular carcinoma (HCC), yet existing models provide limited individualized risk stratification. Automated CT-derived body composition analysis has emerged as an objective marker of patient physiological reserve, but its value in prognostication in TACE patients is insufficiently studied. Therefore, the aim of the study was to evaluate the prognostic value of a fully automated, open-source pipeline for CT-based body composition analysis in predicting overall survival (OS) in patients with HCC undergoing TACE. In this study, we used two independent cohorts of treatment-naive patients undergoing TACE: the WAW-TACE cohort (development; *n* = 230, OS: 28.6 months) and the HCC-TACE-Seg cohort (validation; *n* = 100, OS: 24.0 months). Skeletal muscle and fat metrics were extracted from pre-treatment CTs using a standardized deep learning pipeline and normalized by sex. Survival analyses were performed using Cox proportional hazards (CoxPH) models and random survival forests (RSF). Skeletal muscle density (SMD) at the L3 level was the strongest and independent predictor of OS across both cohorts (HR: development, 0.84; *p* = 0.029; validation, 0.79; *p* = 0.028). This association remained significant after adjustment for the best-performing clinical composite scores: mHAP-2 in the development (adjusted HR = 0.68; *p* = 0.049) and CLIP in the validation cohort (adjusted HR = 0.43; *p* = 0.003). In CoxPH, the addition of SMD metrics resulted in only modest improvements in discrimination (ΔC-index 0.011–0.037) that did not reach statistical significance. In contrast, RSF analysis demonstrated a statistically significant improvement in model discrimination when muscle-based variables were added to clinical features (ΔC-index = 0.023; *p* < 0.001). In both cohorts, SMD showed a reproducible independent prognostic association with overall survival. While adding SMD to traditional clinical models resulted in only modest, and in Cox-based analyses not statistically significant, improvements in discrimination, SMD provided complementary prognostic information. This suggests that the primary value of these automated CT-derived body composition metrics lies not in their performance as standalone predictors, but in their ability to provide an additional layer of objective biological data that may contribute to risk stratification in a complementary and exploratory manner within multivariable frameworks. Notably, in internally cross-validated RSF analyses, statistically significant increases in model discrimination were observed when muscle-based features were integrated into the model, highlighting their potential complementary value within machine learning frameworks.

**Supplementary Information:**

The online version contains supplementary material available at https://doi.org/10.1038/s41598-026-56245-7.

## Introduction

Transarterial chemoembolization (TACE) is a first line, minimally invasive procedure performed primarily in patients with unresectable hepatocellular carcinoma (HCC)^1,2^. Most prognostic scores created for HCC mostly rely on laboratory evaluations of underlying liver disease combined with basic radiologic characteristics, such as the number of tumor lesions and their size^3,4^. Nevertheless, these models are unable to adequately represent the diversity of HCC patients and have limited predictive accuracy^3,5^. Even while two patients may present with tumors of similar size and morphology as well as comparable laboratory parameters, their entire physiological state and, thus, prognosis may differ significantly^6^. As a result, there is a need for more individualized pre-treatment risk-stratification tools that can optimize treatment strategies.

In recent years, the significance of individualized prognostication has been emphasized by a growing number of studies^7,8^. This concept is reflected in the growing interest in frailty-based prognostication approaches^9^. Frailty is understood as reduced physiologic reserve and greater vulnerability to unfavorable treatment outcomes^10^. In clinical practice, frailty is commonly assessed using subjective measures, such as the Clinical Frailty Scale (CFS), which, despite its widespread use, is limited by inter-observer variability^11,12^. In turn, objective body composition analysis has recently been suggested as a scalable alternative for conventional frailty criteria like age or performance status^13^. Variables such as skeletal muscle volume, visceral fat area, and other morphometric indices have been proposed as significant indicators of outcomes in a variety of clinical contexts, including overall survival (OS) in patients with HCC or colorectal cancer^14^. Conventionally, the quantification of these factors relies on the laborious process of manually segmenting regions of interest, which requires hand-crafted input and potentially introduces bias^15^. Therefore, automated segmentation models have gained increasing attention. However, most of the studied models are not open access and are tailored to specific populations, which importantly restricts their wider clinical usage^16^.

A recently published model from Chen et al. enables standardized and fully automated segmentation and extraction of imaging features of muscle and adipose tissue volumes on computed tomography (CT) images, providing a comprehensive body composition profile^13,17^. The pipeline was developed, standardized, and validated on a heterogeneous cohort of CT scans representing multiple patient populations, ensuring generalizability. Recent studies on colorectal cancer patients have demonstrated that features extracted using this method can be utilized for survival prediction and treatment-outcome assessment^14^. However, the prognostic value of automatically derived body composition parameters in patients with HCC, particularly in the setting of TACE, remains insufficiently explored. In this context, body composition features are considered as markers of prognosis within a TACE-treated population rather than as predictors of treatment benefit or as criteria for patient selection.

Therefore, the aim of our study was to evaluate whether body composition parameters derived using a standardized and automated approach may serve as useful prognostic markers for prediction of survival in patients with HCC treated with TACE. For this purpose, we applied a recently released an open-access access model for automated body composition analysis on CT imaging in two independent HCC cohorts.

## Materials and methods

The analysis was performed using data from a previously approved retrospective HCC-TACE-Seg and WAW-TACE datasets. The study was approved by the Bioethics Committee at the Medical University of Warsaw, Warsaw, Poland (decision no. AKBE/41/2024). The requirement for informed consent was waived by the Bioethics Committee at the Medical University of Warsaw due to the retrospective nature of the study.

### Patient cohorts

**Fig. 1:**
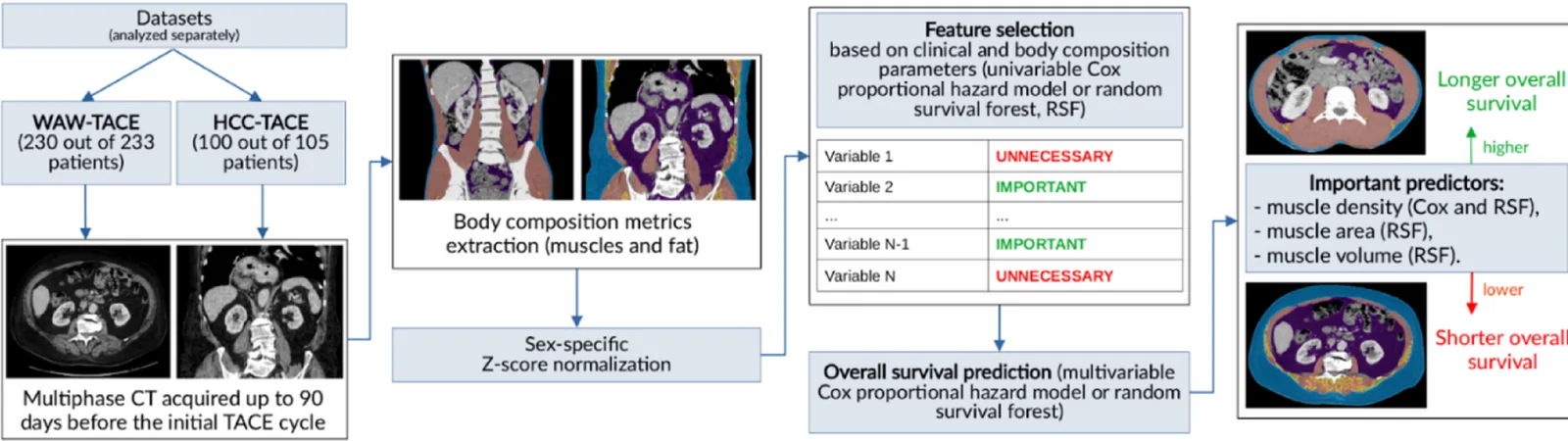
Flowchart of the study. Two independent cohorts (WAW-TACE and HCC-TACE-Seg) were analyzed separately. Following manual quality control to ensure complete L3 coverage and absence of significant artifacts, portal venous phase CT scans acquired prior to TACE were used exclusively for automated extraction of body composition metrics (muscle and fat). This was followed by sex-specific Z-score normalization. Feature selection was performed using clinical and body composition variables based on univariable Cox proportional hazards (CoxPH) models and random survival forests (RSF). Selected features were then incorporated into multivariable survival models for overall survival prediction, showing the prognostic relevance of muscle density, muscle area, and muscle volume.

In this retrospective study, we utilized two independent cohorts: WAW-TACE and HCC-TACE-Seg. The cohorts were selected based on their status as the largest publicly available datasets providing the necessary combination of pre-TACE CT imaging and longitudinal survival outcomes. The WAW-TACE dataset includes 233 patients treated between 2016 and 2021^18^. All patients were treatment-naive and diagnosed with unresectable HCC, subsequently managed with TACE monotherapy. Baseline imaging consists of multiphase contrast-enhanced CT acquired within 90 days prior to the initial TACE cycle. In turn, the HCC-TACE-Seg dataset features 105 patients treated between 2002 and 2012^19^. All cases from this dataset represent confirmed HCC managed with TACE as the sole first-line therapy or as initial bridging therapy. Baseline imaging includes multiphase contrast-enhanced CT performed 1–12 weeks (average 3 weeks) before the first TACE session. Of the original datasets, 3 patients from the WAW-TACE cohort and 5 from the HCC-TACE-Seg cohort were excluded following manual quality control. Reasons for exclusion included incomplete cranio-caudal coverage of the L3 vertebral level and severe motion or metal artifacts interfering with the automated segmentation pipeline (Fig. 1).

### Clinical variables

The clinical variables analyzed included the following:

1. For WAW-TACE dataset: age, sex, alpha-fetoprotein (AFP), total bilirubin, serum albumin, number of tumor lesions, tumor size, Child-Pugh score (CPS), serum creatinine, alanine aminotransferase (ALT), Barcelona Clinic Liver Cancer stage (BCLC), and international normalized ratio (INR). In addition, the following clinical scores were evaluated: ALBI score, mHAP II score, 6&12 score, and HAP score^3,20–22^.
2. For HCC-TACE-Seg dataset: sex, age, CPS, AFP, BCLC, and numeric clinical staging scores including CLIP (Cancer of the Liver Italian Program) score, Okuda, TNM (Tumor-Node-Metastasis)^23,24^.

### Extraction of image-based body composition metrics

All segmentations and subsequent body composition feature extractions were performed exclusively using the portal venous phase of the pre-treatment CT examinations to ensure consistency in tissue attenuation and muscle-fat boundary definition. For the extraction of body composition metrics, we utilized the open-source pretrained deep learning pipeline by Chen et al.^13^. No manual post-processing or correction of the generated muscle and adipose tissue masks was performed to maintain a fully automated workflow. Chen et al. designed an automated, deep learning-based framework capable of segmenting skeletal muscle, visceral adipose tissue, and subcutaneous adipose tissue on axial CT scans of the abdomen and pelvis. The pipeline supports both 2D analysis at the L3 level and 3D volumetric assessment from T12 to L4, providing a comprehensive and standardized set of quantitative measurements, as described in the study by Hanxue et al.^14^.

**Fig. 2:**
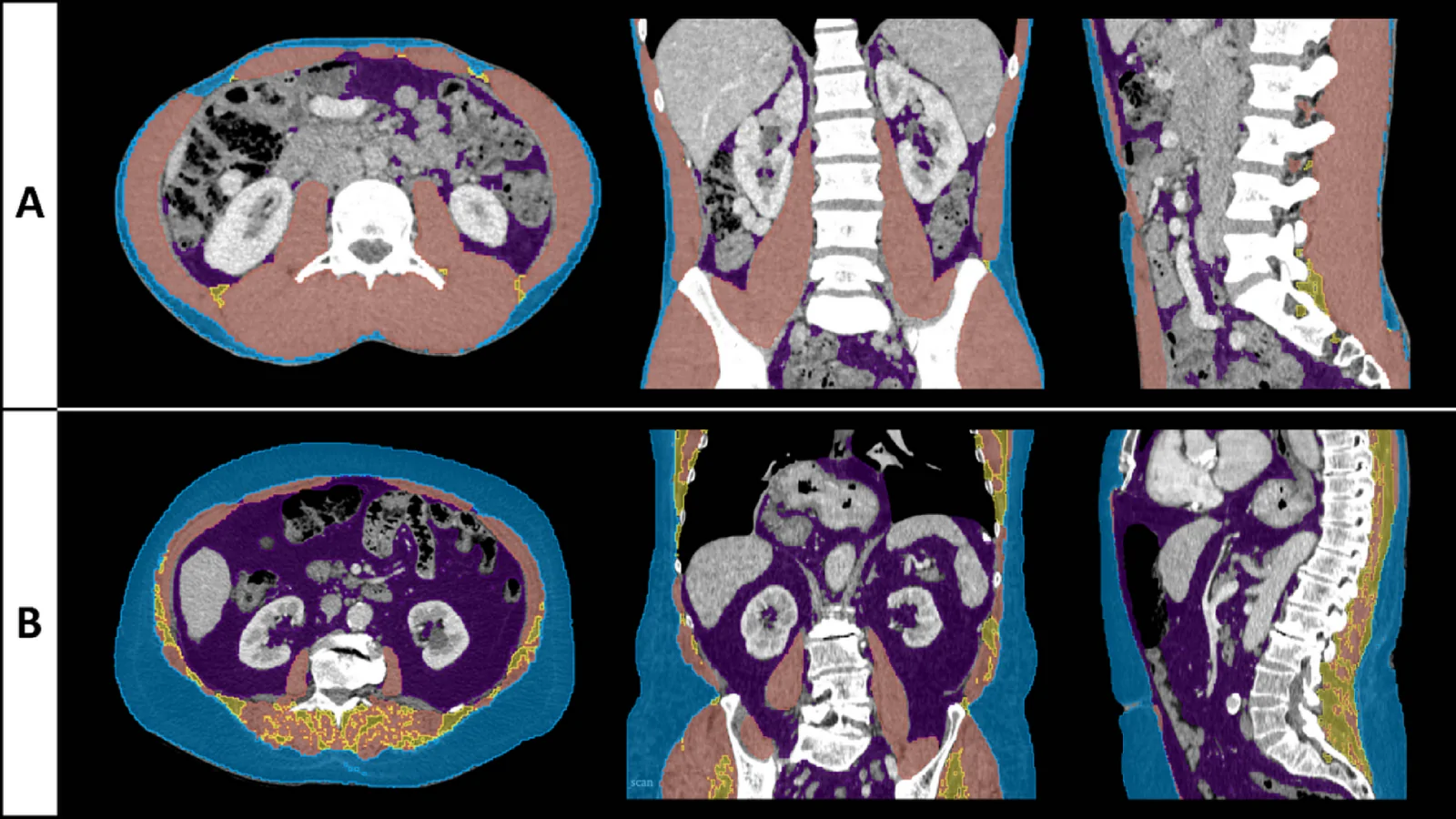
Representative examples of body composition extremes on CT imaging: (**A**) a patient with high skeletal muscle mass and low adipose tissue, and (**B**) a patient with low skeletal muscle mass and high adipose tissue.

The extracted body composition metrics included the following variables. For 2D measurements at the L3 level, these comprised skeletal muscle area (mm²), skeletal muscle density (2D SMD) [HU], subcutaneous fat area (mm²), visceral fat area (mm²), intramuscular fat area (mm²), total fat area (mm²), and total body cross-sectional area (mm²). For 3D measurements between T12 and L4, the extracted variables included skeletal muscle volume (mm³), skeletal muscle density (3D SMD) [HU], subcutaneous fat volume (mm³), visceral fat volume (mm³), intramuscular fat volume (mm³), total fat volume (mm³), total body volume (mm³), and craniocaudal body height between T12 and L4 (mm) (Fig. 2).

### Outcome variables

The primary endpoint of the study was to evaluate the association between automated body composition variables and OS in HCC patients undergoing TACE. Secondary endpoints included 3-year mortality rates and the assessment of whether the addition of CT-derived body-profiling variables could significantly improve the predictive performance of state-of-the-art clinical scores, as well as the best-performing combination of clinical variables.

### Statistical analysis

Quantitative body composition metrics were standardized using sex-specific Z-score normalization to control for sex-related physiological differences that were suggested by previous studies^25^. For the i-th variable, $\:i\in\:\{1,...,N\}$, where * N* is the number of variables, sex-specific means and standard deviations were calculated, and normalized values were derived as Z-scores according to the formula: $\:Z=\frac{x-{\mu\:}_{sex,i}}{{\sigma\:}_{sex,i}}$ where * x* denotes the observed value, while $\:{\mu\:}_{sex,i}$ and $\:{\sigma\:}_{sex,i}$ represent the sex-specific mean and standard deviation for the i-th variable, respectively. 2D SMD was dichotomized using a data-driven cut-off, resulting in a binary muscle density indicator (binary 2D SMD). The classification into low and high subgroups in the Kaplan-Meier analysis was performed using an optimal cut-off value, determined by maximizing the log-rank test statistic (Contal & O’Quigley-like method)^26^.

Body-composition parameters and clinical variables were first evaluated using univariable Cox proportional hazards (CoxPH) analyses. Variables that reached statistical significance (*p* < 0.05) in the univariable analysis were subsequently included in multivariable CoxPH models to assess their independent predictive value. The concordance index (C-index) was used to evaluate the predictive performance of the CoxPH models for OS. Additionally, the area under the ROC curve (AUC) was calculated for 3-year mortality. Performance differences between models were quantified using ΔC-index and ΔAUC(t), with bootstrap-based confidence intervals.

Additionally, a machine learning-based approach was used to assess the impact of body composition parameters on OS prediction. Firstly, a 5-fold cross-validation (CV) experiment using Random Survival Forests (RSF) with all available variables and 100 estimators was performed. In each cross-validation step, a permutation-based feature importance was calculated. After five CV folds, variables having a positive mean importance score, i.e. causing a more accurate C-index estimation, were selected. Then, the RSF model with variables considered important in the previous step (both clinical and body composition) was compared with the model trained using only a significant subset of clinical variables. This evaluation was performed in another 5-fold cross-validation experimental scenario with 10 repetitions. Finally, to assess the significance of the difference in model performance, a t-test for related samples was performed. All RSF comparisons were performed within internal cross-validation; therefore, these results should be interpreted as supportive evidence of incremental value in a machine learning setting rather than definitive evidence of clinical utility.

All statistical analyses and figure generation were performed in Python 3.13.0 (Python Software Foundation 2024, Language Reference Version 3.13.0, https://www.python.org/) in PyCharm 2025.3.1 (JetBrains s.r.o.; https://www.jetbrains.com/pycharm/). Data handling was conducted with the *pandas* and *NumPy* libraries. Survival analyses were performed using the *lifelines* and *scikit-survival* packages, including Cox proportional hazards regression (*CoxPHFitter*) and calculation of Harrell’s concordance index. Standard Python utilities (*os*, *pathlib*) and typing modules were used for file handling and code organization. Analyses were executed in a reproducible Python environment, and the complete source code and environment specifications can be provided upon reasonable request.

## Results

### Characteristics of patient populations

WAW-TACE dataset: the final analysis included 230 patients who underwent TACE and had an available portal venous phase. OS was 28.6 months (95% CI: 21.8–33.8); 1-year and 3-year mortality rates were 26.5% and 59.7%, respectively.

HCC-TACE-Seg dataset: 100 patients who underwent TACE and had an available portal venous phase were included. OS was 24.0 months (95% CI: 19.6–29.0). The 1-year and 3-year mortality rates were 22.1% and 73.6%, respectively.

A detailed characterization of both evaluated cohorts is presented in Table 1.

**Table 1:** Baseline clinical characteristics of patients included in the study in the WAW-TACE and HCC-TACE-Seg cohorts.

Variable	WAW-TACE (*n* = 230)	HCC-TACE-Seg (*n* = 100)	*p* value
Female sex, n (%)	48 (20.9)	35 (35.0)	0.01
Age, median (years)	66	68.5	0.17
Child-Pugh class, n (%)			0.08
A	204 (88.7)	81 (81.0)	
B	26 (11.3)	18 (18.0)	
C	-	1 (1.0)	
AFP, n (%)			0.46
< 400 ng/mL	176 (76.5)	72 (72.0)	
≥ 400 ng/mL	54 (23.5)	28 (28.0)	
AFP, median (ng/mL)	14.25	32.9	0.15
BCLC stage, n (%)			< 0.01
0/A	168 (73.0)	15 (15.0)	
B	62 (27.0)	23 (23.0)	
C	-	62 (62.0)	

Continuous variables are reported as medians and categorical variables as counts with percentages. Differences between cohorts were assessed using appropriate statistical tests for continuous and categorical variables, with corresponding *p* values reported.

### WAW-TACE

#### Univariable survival analysis

Full univariate CoxPH results for all raw and sex-normalized fat and muscle-based predictors are provided in the Supplementary Tables S1 and S2, respectively. All subsequent muscle- and fat-related variables reported in the following sections refer to these sex-normalized measures. Among all image-derived variables, only 2D SMD and 3D SMD were significant predictors of OS. The corresponding hazard ratios were 0.84 (95% CI, 0.72–0.98; *p* = 0.029) for 2D SMD and 0.85 (95% CI, 0.73-1.00; *p* = 0.045) for 3D SMD. Among clinical variables, serum albumin, tumor size, log-transformed AFP, total bilirubin, number of tumor lesions, and Child-Pugh score were significant predictors of OS (Supplementary Table S3). Among all variables evaluated in the univariable analysis, the mHAP-2 score was the strongest predictor of OS (HR 1.58, 95% CI 1.32–1.89, *p* < 0.01) with the lowest C-index of 0.641 (Supplementary Table S3).

#### Determination of binary indicator for skeletal muscle density at the L3 level

**Fig. 3:**
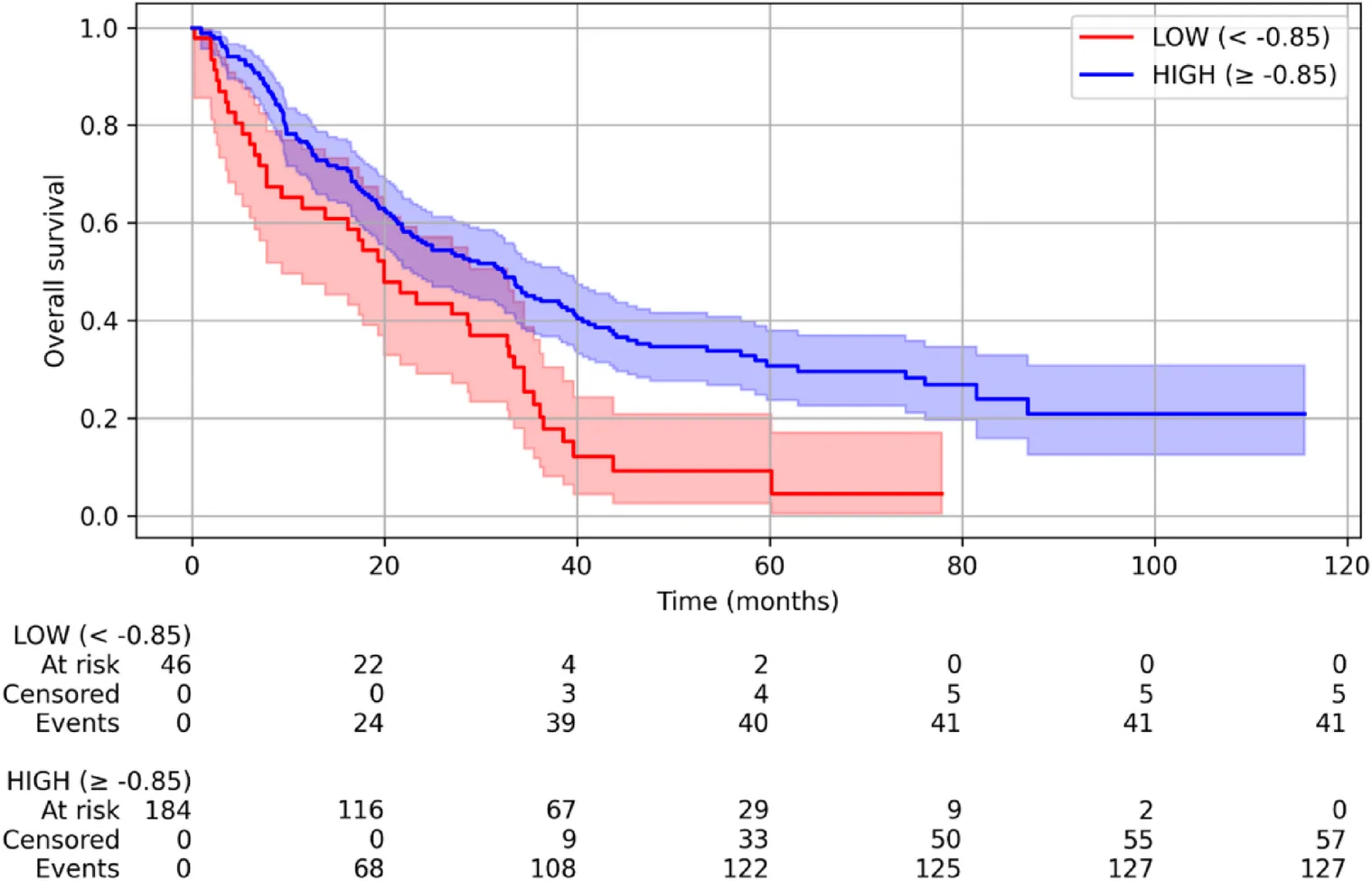
Kaplan-Meier curves for overall survival stratified by the binary indicator for skeletal muscle density at the L3 level in the WAW-TACE cohort. Patients were dichotomized into high and low muscle-density groups using an optimal cut-off derived from a Contal and O’Quigley-type maximally selected log-rank statistic. Patients with low skeletal muscle density are shown in red, whereas those with high skeletal muscle density are shown in blue. Numbers at risk are displayed below the plot.

In the cut-off selection procedure for 2D SMD, the optimal threshold was identified as -0.85 with the log-rank test *p* < 0.001 (Fig. 3). This binary SMD indicator was subsequently evaluated in univariate survival models. For OS, the binary SMD was significantly associated with mortality risk (HR = 0.53, 95% CI: 0.37–0.76, *p* = 0.0005). For 3-year mortality, the association was also significant (HR = 0.59, 95% CI: 0.40–0.86, *p* = 0.006). Patients below the cut-off had a median OS of 19.9 months (95% CI: 9.3–32.8), whereas those above the cut-off had a median OS of 32.3 months (95% CI: 22.9–38.4). At 3 years, survival rates were 22.8% (95% CI: 11.8–36.0) and 44.5% (95% CI: 37.2–51.5), respectively.

#### Multivariable analysis

After adjusting for the mHAP-2 score (the strongest clinical composite score associated with OS), binary 2D SMD remained independently associated with OS (HR 0.68, 95% CI 0.46-1.00, *p* = 0.049). When evaluated as a predictive marker, adding binary 2D SMD to the mHAP-2 model resulted in only a small improvement in discrimination: the C-index increased from 0.641 to 0.657 (Δ = 0.015; 95% CI -0.006 to 0.039; *p* = 0.513). A similar modest gain was observed when adding continuous 2D SMD instead of the binary cut-off (C-index 0.657; Δ = 0.016; 95% CI -0.001 to 0.033; *p* = 0.485).

Similar results were obtained in an analysis adjusting for individual clinical variables rather than composite clinical scores. Binary 2D SMD remained an independent predictor after adjustment for state-of-the-art clinical confounders identified in univariable analysis (Supplementary Table S4), including serum albumin, total bilirubin, log-transformed AFP, Child-Pugh score, tumor size, and number of tumor lesions. Incorporation of binary 2D SMD to clinical variables resulted in a modest improvement in model discrimination, with the concordance index increasing from 0.672 for the clinical model to 0.684 for the extended model (ΔC-index = 0.011, 95% CI − 0.003 to 0.027; bootstrap *p* = 0.50).

A similar pattern was observed in time-specific discrimination. At 3 years, the time-dependent AUC increased from 0.698 for the clinical-only model to 0.720 after inclusion of binary 2D SMD (ΔAUC = 0.022); however, this improvement did not reach statistical significance (95% CI, − 0.002 to 0.046; bootstrap *p* = 0.50). A comparable improvement was observed when a continuous 2D SMD was used instead (ΔAUC = 0.026, *p* = 0.51).

#### Machine learning-based feature importance estimation

**Fig. 4:**
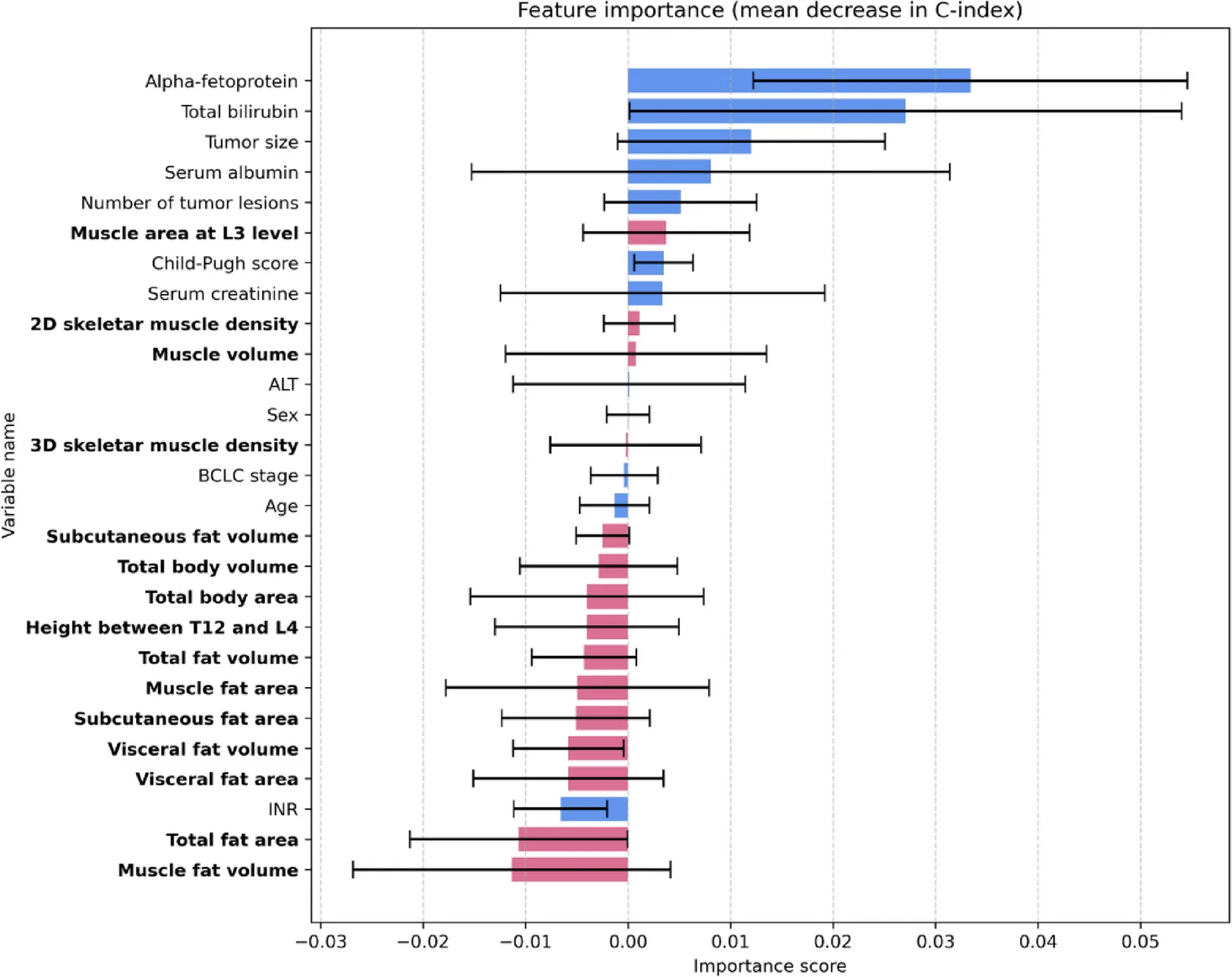
Permutation feature importance analysis showing the mean decrease in concordance index for clinical variables (blue) and muscle-fat imaging features (red, shown in bold). Higher values indicate a greater contribution of the corresponding feature to model performance.

The feature importance estimation with RSF revealed that 9 clinical and 3 muscle-based variables are valuable predictors with positive importance scores (Fig. 4). The most important variables were log-transformed AFP, total bilirubin, and tumor size, belonging to the category of clinical variables. The three selected muscle-based predictors, i.e. skeletal muscle area at the L3 level, 2D SMD, and skeletal muscle volume, were considered to have a positive influence on model estimations. The remaining 15 variables were excluded from further calculations.

In the second cross-validation stage, a model with a subset of all variables was compared with a model having only a subset of clinical predictors. The combined model obtained the C-index of 0.6578, while the clinical-only model achieved 0.6352. Since the differences in predictions between the two models were normally distributed according to the results of the Shapiro-Wilk test (W = 0.98, *p* = 0.59), the one-sided t-test for related samples was calculated. Its results (t = 6.04, *p* < 0.001) indicate that the inclusion of muscle-based predictors together with clinical variables resulted in a statistically significant improvement in model performance compared with the clinical-only model.

### HCC-TACE-seg

#### Univariable survival analysis

Full univariate CoxPH results for all sex-normalized fat and muscle metrics are provided in the Supplementary Table S5. Among all evaluated factors, 2D SMD and 3D SMD were the only variables significantly associated with OS in the univariable analysis, which was analogous to results on the WAW-TACE dataset. The corresponding hazard ratios were 0.79 (95% CI, 0.65–0.98; *p* = 0.028) for 2D SMD and 0.80 (95% CI, 0.65–0.99; *p* = 0.045) for 3D SMD.

#### Determination of binary indicator for skeletal muscle density at the L3 level

In the cut-off selection procedure for 2D SMD, the optimal threshold was identified as 0.61, which yielded a statistically significant separation in survival curves (log-rank *p* = 0.002). The dichotomized variable was subsequently evaluated in univariable CoxPH, demonstrating a strong association with overall survival (HR = 0.43, 95% CI: 0.25–0.75, *p* = 0.0029). The median OS in the cohort was 24.0 months (95% CI: 19.6–29.0). Patients classified as having low 2D SMD according to the binary indicator had a median OS of 24.0 months (95% CI: 17.5–28.1), whereas those classified as having high 2D SMD had a median OS of 35.6 months (95% CI: 15.2–81.6). At 3 years, OS was 18.6% (95% CI: 10.6–28.5) and 48.0% (95% CI: 27.8–65.6), respectively.

**Fig. 5:**
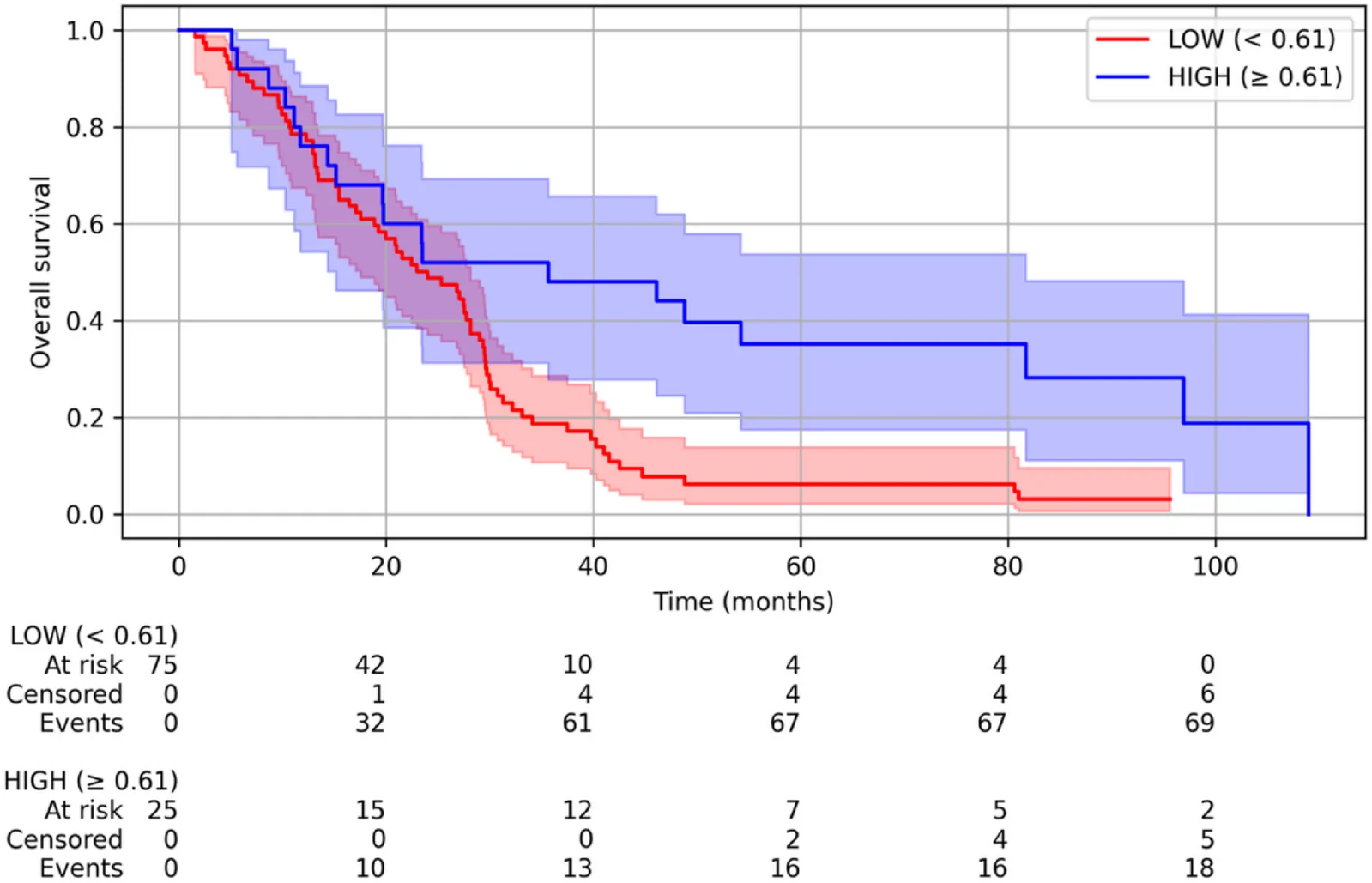
Kaplan-Meier curves for overall survival stratified by the binary indicator for skeletal muscle density at the L3 level in the HCC-TACE-Seg cohort. Patients were dichotomized into high and low muscle density groups using an optimal cut-off derived from a Contal and O’Quigley-type maximally selected log-rank statistic. Patients with low skeletal muscle density are shown in red, whereas those with high skeletal muscle density are shown in blue. Numbers at risk are displayed below the plot.

#### Multivariable analysis

Within this dataset, the CLIP score emerged as the strongest clinical predictor of mortality (HR 1.43, 95% CI 1.15–1.77, *p* = 0.0012) (Supplementary Table S6). In the multivariable CoxPH model, including the CLIP score and binary 2D SMD, both covariates were significantly associated with OS. Binary 2D SMD showed a hazard ratio of 0.42 (95% CI: 0.24–0.73, *p* = 0.002).

In the multivariable CoxPH analysis for OS, incorporation of the binary 2D SMD into the clinical model based on the CLIP score resulted in a numerically higher discriminative performance. Specifically, the C-index increased from 0.565 for the clinical-only model to 0.602 for the extended model including the binary 2D SMD, corresponding to an absolute ΔC-index of 0.037. However, this improvement did not reach statistical significance (95% CI, − 0.018 to 0.094; bootstrap *p* = 0.498). At 3 years, time-dependent discrimination improved from an AUC of 0.579 to 0.704 (ΔAUC = 0.125). While the bootstrap confidence interval suggested a non-zero effect (95% CI, 0.019–0.234), bootstrap-based hypothesis testing did not indicate statistical significance (*p* = 0.466).

## Discussion

In this study, we evaluated the prognostic significance of standardized CT-derived body composition parameters on survival in patients with unresectable HCC treated with TACE. The results were validated in two independent datasets, supporting their robustness and generalizability, which is a major strength of this study. Despite differences in sample size, timeframe, and patient characteristics across both cohorts, SMD consistently showed a significant independent association with OS. The reproducibility of this finding across two different clinical populations suggests that muscle quality captures important aspects of physiological reserve and patient frailty that traditional clinical scores do not fully capture. Specifically, in the WAW-TACE cohort, 2D SMD and 3D SMD demonstrated an association with survival, and the effect of 2D SMD remained preserved even after adjustment for established clinical scores such as the mHAP-2 score. Similarly, in the HCC-TACE-Seg dataset, both muscle density measurements (2D SMD and 3D SMD) were significantly associated with mortality risk, reinforcing the robustness of this metric across different clinical settings. Most other body composition variables, including fat-related metrics and muscle size measures, did not show consistent significant associations with survival across cohorts. At the same time, our evaluation of predictive performance showed that although muscle density remained independently associated with mortality in multivariable CoxPH models, its incremental contribution to overall model discrimination was modest and did not reach statistical significance in Cox-based comparisons. For example, in the WAW-TACE cohort, adding binary 2D SMD to a combination of significant clinical variables increased the C-index only slightly (ΔC-index = 0.011), and a similar limited improvement was observed in the 3-year AUC (ΔAUC = 0.022). Although the inclusion of muscle density consistently yielded numerically higher C-indices, the bootstrap CI crossed zero and the differences were not statistically significant, indicating that the observed improvements in discrimination were modest and likely reflect a small incremental prognostic value. These findings further suggest that the study may be underpowered to detect small but statistically significant differences in model performance. Importantly, in internally cross-validated RSF analyses, a model incorporating a subset of both clinical and muscle-based variables achieved a significantly higher performance than the clinical-only model (ΔC-index = 0.023; *p* < 0.001). Given that all patients in both cohorts were treated with TACE, the observed associations should be interpreted as prognostic and not as evidence of treatment benefit or as guidance for patient selection.

These findings indicate that automated CT-based body composition analysis including muscle density assessment provides prognostically relevant information not fully captured by standard clinical variables, but the magnitude of improvement in model performance remains relatively small. In other words, automated skeletal muscle density may be valuable as a complementary and exploratory imaging-derived biomarker within broader multivariable prognostic frameworks, rather than as a standalone predictor intended to substantially enhance discrimination. In particular, muscle density may be especially useful in machine learning-based and radiomics-driven approaches. Those models frequently rely on multiple imaging features, where SMD can serve as an additional biologically interpretable variable contributing incremental prognostic value and improving overall model performance^27^. Rather than acting as standalone predictors, these features function as complementary biological layers that enhance the depth of discriminative frameworks, particularly when subtle shifts in metabolic health impact survival outcomes independently of tumor stage. Therefore, its integration into imaging-only models warrants further investigation.

Our results align with previous reports linking reduced muscle quality to poorer outcomes following colorectal surgery. A recent study by Hanxue Gu et al. showed that sex-normalized SMD -measured in the same way as in our study - was a strong and independent predictor of 1-year mortality after colectomy (OR 0.42)^14^. The authors showed that these metrics have prognostic value independent of traditional clinical variables and enhance the discriminatory accuracy of established risk calculators (ΔC-index = 0.07, 0.80 vs. 0.73), whereas in our study, they improved prediction only modestly (ΔC-index values ranging from 0.011 to 0.037). Importantly, our similar observations in an entirely different clinical context support the idea that automated SMD represents a reliable biomarker that generalizes across a variety of clinical scenarios and imaging protocols. This implies that automated CT-based muscle quality assessment captures significant parts of patient frailty and physiologic reserve that are not represented by conventional clinical variables. As a result, these measures could be useful as adjuncts for individualized prognostic assessment in a variety of patient groups, although this requires further validation. This method is easy to implement and does not require additional procedures beyond standard clinical care. Once included in the clinical workflow, the model can be fully automated.

These findings are consistent with a recent meta-analysis that summarizes the predictive significance of body composition in HCC patients. The pooled HR = 1.38 for sarcopenia as a predictor of OS in TACE patients, suggesting that subjects with low muscle quality have a significantly higher mortality^15^. However, the methodologies used to assess muscle mass and muscle quality differed substantially across the included studies, ranging from manual and semi-automated segmentations at different anatomical levels to threshold-based approaches and ROI measurements. This variability makes direct comparison more difficult, restricts wider clinical translation, and underscores the need for standardized, reproducible approaches to body composition evaluation. In this context, the fully automated and publicly available model used in our study offers a potential solution by providing consistent and observer-independent measurements that may facilitate standardization across centers and imaging protocols.

A conceptually similar automated approach was previously presented by Wang et al., who developed an in-house deep convolutional neural network to facilitate psoas muscle segmentation on CT studies^16^. Their study served primarily as a proof-of-concept that deep learning can replace manual measurements of psoas muscle volume and that such an automated psoas muscle index is independently associated with OS in patients with liver cirrhosis. A cohort of 254 people with chronic liver disease was used for clinical validation in their investigation. The study showed that automated psoas size measurement was a significant survival predictor even after adjusting for age, sex, and liver function. Specifically, the CT-derived psoas muscle index was significantly associated with survival (HR = 0.86), but the study did not present parametric model performance metrics comparing models with and without this parameter. Moreover, the model used in that study was trained and assessed mostly on internal datasets, lacked external validation, and was not made available as a standardized, publicly accessible tool, that restricts its wider clinical applicability. Despite limitations, these results offered proof that prognostic information is carried by image-based muscle measures. Our study expands this concept beyond chronic liver disease in general to a more clinically specific context: TACE patients with HCC. Additionally, our analysis relied on an open-source, pretrained, fully automated pipeline that, once applied, does not require manual annotation or additional radiologist input, unlike Wang et al.‘s approach. This substantially lowers the barrier to integration in other clinical centers and supports the feasibility of using standardized body-composition profiling as part of routine pre-TACE risk stratification.

A series of related observations was reported by Surov et al., who studied the prognostic role of muscle quality in patients with HCC treated with different locoregional therapies. In one study, involving patients undergoing combined SIRT and sorafenib treatment, skeletal muscle density was measured semi-automatically at the L3 level with manual radiologist corrections^28^. Myosteatosis was associated with OS in the univariable analysis, although this effect did not remain significant after adjustment for demographic and clinical variables. Notably, subgroup analyses showed that myosteatosis was a significant predictor only in patients with alcohol-related cirrhosis, suggesting that its importance may depend on underlying liver disease. In another study by the same authors, using a similar semi-automated methodology in cirrhotic patients treated with TACE, myosteatosis again did not demonstrate an independent association with survival, whereas the albumin-muscle density score emerged as a significant predictor^29^. Both studies reported associations with survival but did not include a formal evaluation of the additive model performance of imaging-derived muscle parameters beyond hazard ratios.

The broader prognostic relevance of body composition measurements has been demonstrated across multiple clinical settings. Sarcopenia and myosteatosis have been associated with increased mortality in patients with breast cancer, chronic liver disease, chronic kidney disease, and liver transplantation^30–34^. Collectively, these results suggest that muscle mass and muscle quality are significant indicators of physiological susceptibility in oncology and other medical scenarios. This supports the idea that a fully automated, standardized CT-based methodology, like the one employed in our study, could be potentially applicable in a variety of illness scenarios that require individualized risk stratification.

### Limitations

This study has several limitations that should be acknowledged. First, the retrospective design of both cohorts introduces the possibility of selection bias and unmeasured confounding that may influence outcomes independently of body composition metrics. Second, although the use of two independent datasets enhances the generalizability of our findings, the cohorts differed with respect to treatment timeframe (2002–2012 vs. 2016–2021), clinical practice standards, imaging protocols, scanner types, and baseline patient characteristics. These sources of heterogeneity, combined with the lack of scanner-specific normalization, may affect the comparability of absolute muscle-density values and the magnitude of observed associations. Thirdly, the observed heterogeneity in baseline characteristics between the WAW-TACE and HCC-TACE-Seg cohorts - specifically the higher prevalence of BCLC stage A in the former and stage C in the latter - represents a significant challenge for model generalizability. However, the fact that body composition metrics, particularly muscle density, maintained prognostic relevance across these diverse stages suggests that metabolic health remains a critical survival determinant regardless of tumor burden. Moreover, although the use of independent datasets provides external validation, the inherent differences in disease stage distribution and clinical practices between them may limit the direct generalizability of our specific C-index values. Future prospective studies with more uniform inclusion criteria are needed to establish standardized prognostic thresholds. Furthermore, our analysis was limited to baseline CT examinations. However, it is worth noting that longitudinal changes in body composition during treatment also play a critical role in prognosis. Recent evidence suggests that treatment-related cachexia is associated with poorer clinical outcomes in HCC patients^35^. Incorporating serial assessments to monitor these dynamic changes remains an important direction for future research. Additionally, the absence of a control group not receiving TACE limits our ability to distinguish between general prognostic factors and those specific to TACE response. Our findings should therefore be interpreted as prognostic within the treated population rather than predictive of treatment effect. Finally, the optimal cut-off for muscle density was derived using maximally selected log-rank statistics, which carries an inherent risk of overfitting. These data-driven cut-offs are exploratory and cohort-specific, as reflected by differing thresholds between the development and validation cohorts, and may overestimate effect sizes, thus requiring independent, preferably prospective studies before clinical application.

### Future directions

To further expand these findings, future research can potentially focus on filling gaps in knowledge. First, a more comprehensive “combined” approach that captures both patient- and tumor-specific prognostic variables could be achieved by combining body composition measures with ML-based tumor assessment. Second, to develop clinically relevant thresholds for muscle and fat measures across different populations, prospective, multi-center studies are required. Lastly, examining the role of longitudinal body composition evaluations throughout TACE therapy may provide important information about treatment-related body adaptations and its influence on patient survival.

## Conclusions

In patients with unresectable HCC treated with TACE, automated, quantitative CT-based assessment of skeletal muscle density emerged as an independent predictor of OS. This effect was reproducible in two independent cohorts and consistent with findings from an external colorectal cancer study, supporting potential broader relevance beyond HCC. Although its addition led to only modest improvements in discrimination over established clinical models, the automated muscle-density measure is easy to implement, scalable, and may serve as a useful complementary imaging biomarker within multimodal prognostic frameworks. Any potential clinical integration would require independent, preferably prospective validation, and the present results should not be interpreted as establishing clinical actionability, treatment-selection utility, or established patient-selection value.

## Supplementary Information

Below is the link to the electronic supplementary material.


Supplementary Material 1


## Data Availability

All datasets used in this study are publicly available. The WAW-TACE dataset is available in the Radiology: Artificial Intelligence under DOI: 10.1148/RYAI.240296. The HCC‑TACE‑Seg dataset is available in the Scientific Data under DOI: 10.1038/s41597‑023‑01928‑3.
