# Supplementary material for: Fully automated CT-based quantitative body composition analysis for predicting survival in patients with HCC undergoing TACE: a dual-cohort study
Source: Sci Rep. 2026 Jun 16;16:27444. doi: 10.1038/s41598-026-56245-7 (PMC13534685; doi:10.1038/s41598-026-56245-7)
Supplement: Supplementary file 1 [file 41598_2026_56245_MOESM1_ESM.docx]

**Fully Automated CT-Based Quantitative Body Composition Analysis for Predicting Survival in Patients with HCC Undergoing TACE: A Dual-Cohort Study**

Authors: Krzysztof Bartnik^1,2^, Belgutei Tsevegmed^1^, Kamil Książek^2^, Zuzanna Wojtczak*^1^, Przemysław Biecek^2^

1. Second Department of Radiology, Medical University of Warsaw, Warsaw, Poland
2. Centre for Credible Artificial Intelligence, Warsaw University of Technology, Warsaw, Poland

*Corresponding author: Zuzanna Wojtczak MD, zuzanna.wojtczak@wum.edu.pl, cell phone: +48 22 599 23 00; Banacha 1a st, 02-097 Warsaw, Poland.

**Supplementary Materials**

Contents

[WAW-TACE	1](#_Toc220432881)

[Supplementary Table S1. Univariable Cox analysis of muscle and fat variables using raw (non–sex-normalized) data.	1](#_Toc220432882)

[Supplementary Table S2. Univariable Cox analysis of muscle and fat metrics after sex normalization.	2](#_Toc220432883)

[Supplementary Table S3. Univariable Cox analysis of clinical predictors of overall survival.	3](#_Toc220432884)

[Supplementary Table S4. Multivariable Cox regression analysis for overall survival including clinical variables and the binary muscle density indicator (at L3 level).	4](#_Toc220432885)

[HCC-TACE-SEG	5](#_Toc220432886)

[Supplementary Table S5. Univariable Cox analysis of muscle and fat metrics after sex normalization.	5](#_Toc220432887)

[Supplementary Table S6. Univariable Cox analysis of clinical predictors of overall survival.	6](#_Toc220432888)

# WAW-TACE

Supplementary Table S1. Univariable Cox analysis of muscle and fat variables using raw (non–sex-normalized) data.

*HR* hazard ratio; *95% CI* 95% confidence interval*;* 2D metrics are measured at the L3 level (areas, densities), 3D metrics between T12 and L4 (volumes, densities).

| Variable | HR | 95% CI (Lower) | 95% CI (Upper) | *p* value |
| --- | --- | --- | --- | --- |
| Average 2D muscle density | 0.98 | 0.97 | 1.00 | 0.04 |
| Average 3D muscle density | 0.99 | 0.97 | 1.00 | 0.05 |
| Total body volume | 1.00 | 1.00 | 1.00 | 0.13 |
| Height between T12 and L4 | 1.00 | 1.00 | 1.01 | 0.17 |
| Total body area | 1.00 | 1.00 | 1.00 | 0.42 |
| Muscle fat area | 1.00 | 1.00 | 1.00 | 0.44 |
| Subcutaneous fat volume | 1.00 | 1.00 | 1.00 | 0.45 |
| Muscle volume | 1.00 | 1.00 | 1.00 | 0.51 |
| Muscle area at L3 level | 1.00 | 1.00 | 1.00 | 0.64 |
| Total fat volume | 1.00 | 1.00 | 1.00 | 0.65 |
| Visceral fat area | 1.00 | 1.00 | 1.00 | 0.70 |
| Subcutaneous fat area | 1.00 | 1.00 | 1.00 | 0.79 |
| Visceral fat volume | 1.00 | 1.00 | 1.00 | 0.92 |
| Total fat area | 1.00 | 1.00 | 1.00 | 0.93 |
| Muscle fat volume | 1.00 | 1.00 | 1.00 | 0.95 |

Supplementary Table S2. Univariable Cox analysis of muscle and fat metrics after sex normalization.

*HR* hazard ratio; *95% CI* 95% confidence interval*; 2*D metrics are measured at the L3 level (areas, densities), 3D metrics between T12 and L4 (volumes, densities).

| Variable | HR | 95% CI (Lower) | 95% CI (Upper) | *p* value |
| --- | --- | --- | --- | --- |
| Binary muscle density indicator (at L3 level) | 0.53 | 0.37 | 0.76 | <0.01 |
| Average 2D muscle density | 0.84 | 0.72 | 0.98 | 0.03 |
| Average 3D muscle density | 0.85 | 0.73 | 1.00 | 0.05 |
| Muscle area at L3 level | 0.88 | 0.75 | 1.04 | 0.15 |
| Visceral fat area | 0.93 | 0.80 | 1.08 | 0.36 |
| Muscle fat area | 1.06 | 0.91 | 1.23 | 0.45 |
| Subcutaneous fat volume | 1.06 | 0.90 | 1.24 | 0.48 |
| Total body volume | 1.06 | 0.90 | 1.25 | 0.50 |
| Muscle volume | 0.94 | 0.79 | 1.13 | 0.53 |
| Visceral fat volume | 0.96 | 0.82 | 1.13 | 0.63 |
| Subcutaneous fat area | 1.03 | 0.88 | 1.21 | 0.68 |
| Total body area | 1.03 | 0.88 | 1.20 | 0.70 |
| Height between T12 and L4 | 1.04 | 0.86 | 1.25 | 0.70 |
| Total fat area | 0.97 | 0.83 | 1.13 | 0.71 |
| Muscle fat volume | 0.99 | 0.85 | 1.15 | 0.92 |
| Total fat volume | 1.00 | 0.85 | 1.17 | 0.99 |

Supplementary Table S3. Univariable Cox analysis of clinical predictors of overall survival.

*HR* hazard ratio; *95% CI* 95% confidence interval*; C-index* concordance index; *Tumor size* tumor size categorized at 0/6/12 cm; *AFP (log)* log-transformed α-fetoprotein; *AFP (continuous)* untransformed continuous α-fetoprotein; *CPS* Child-Pugh Score; *ALT* alanine aminotransferase; *BCLC* Barcelona Clinic Liver Cancer stage; *INR* international normalized ratio; *ALBI score* Albumin–Bilirubin score; *mHAP II score* the modified Hepatoma Arterial-embolization Prognostic II score; *HAP score* the Hepatoma Arterial-embolization Prognostic score.

| Variable | HR | 95% CI (Lower) | 95% CI (Upper) | *p* value | C-index |
| --- | --- | --- | --- | --- | --- |
| Serum Albumin | 0.50 | 0.37 | 0.67 | <0.01 | - |
| Tumor size | 1.85 | 1.41 | 2.42 | <0.01 | - |
| AFP (log) | 1.29 | 1.15 | 1.45 | <0.01 | - |
| Total Bilirubin | 1.34 | 1.14 | 1.56 | <0.01 | - |
| AFP (continuous) | 1.00 | 1.00 | 1.00 | <0.01 | - |
| ALBI score | 2.03 | 1.66 | 2.48 | <0.01 | 0.635 |
| mHAP II score | 1.59 | 1.37 | 1.84 | <0.01 | 0.641 |
| 6&12 score | 1.19 | 1.13 | 1.26 | <0.01 | 0.580 |
| HAP score | 1.62 | 1.38 | 1.90 | <0.01 | 0.637 |
| Number of Tumor Lesions (≥3) | 1.97 | 1.16 | 3.35 | 0.01 | - |
| CPS (B) | 1.75 | 1.12 | 2.75 | 0.01 | - |
| Number of Tumor Lesions (≥4) | 1.93 | 1.08 | 3.44 | 0.03 | - |
| Sex (Female) | 0.74 | 0.50 | 1.09 | 0.12 | - |
| ALT | 1.00 | 1.00 | 1.00 | 0.16 | - |
| Serum Creatinine | 0.66 | 0.35 | 1.22 | 0.19 | - |
| BCLC Stage (B/C) | 1.18 | 0.85 | 1.65 | 0.33 | - |
| INR | 1.40 | 0.57 | 3.43 | 0.46 | - |
| Age | 1.00 | 0.99 | 1.01 | 0.95 | - |
| Number of Tumor Lesions (2) | 1.01 | 0.69 | 1.47 | 0.98 | - |

Supplementary Table S4. Multivariable Cox regression analysis for overall survival including clinical variables and the binary muscle density indicator (at L3 level).

*HR* hazard ratio; *95% CI* 95% confidence interval*; AFP (log)* log-transformed α-fetoprotein; *CPS* Child-Pugh Score.

| Variable | HR | 95% CI (Lower) | 95% CI (Upper) | *p* value |
| --- | --- | --- | --- | --- |
| Serum Albumin | 0.59 | 0.42 | 0.84 | <0.01 |
| AFP (log) | 1.22 | 1.08 | 1.37 | <0.01 |
| 6&12 score = 2 | 4.69 | 2.28 | 9.63 | <0.01 |
| Binary muscle density indicator (at L3 level) | 0.61 | 0.42 | 0.90 | 0.01 |
| Total Bilirubin | 1.33 | 1.04 | 1.70 | 0.02 |
| Number of Tumor Lesions (≥4) | 1.73 | 0.93 | 3.21 | 0.08 |
| 6&12 score = 1 | 1.30 | 0.93 | 1.82 | 0.12 |
| Number of Tumor Lesions (≥3) | 1.50 | 0.84 | 2.67 | 0.17 |
| CPS (B) | 0.60 | 0.28 | 1.30 | 0.20 |
| Number of Tumor Lesions (2) | 1.04 | 0.71 | 1.54 | 0.83 |

# HCC-TACE-SEG

Supplementary Table S5. Univariable Cox analysis of muscle and fat metrics after sex normalization.

*HR* hazard ratio; *95% CI* 95% confidence interval*;* 2D metrics are measured at the L3 level (areas, densities), 3D metrics between T12 and L4 (volumes, densities).

| Variable | HR | 95% CI (Lower) | 95% CI (Upper) | *p* value |
| --- | --- | --- | --- | --- |
| Binary muscle density indicator (at L3 level) | 0.43 | 0.25 | 0.75 | <0.01 |
| Average 2D muscle density | 0.79 | 0.65 | 0.98 | 0.03 |
| Average 3D muscle density | 0.80 | 0.65 | 0.99 | 0.04 |
| Height between T12 and L4 | 0.85 | 0.68 | 1.07 | 0.16 |
| Muscle volume | 0.86 | 0.68 | 1.09 | 0.21 |
| Total body area | 1.11 | 0.90 | 1.38 | 0.32 |
| Muscle fat area | 1.10 | 0.86 | 1.39 | 0.45 |
| Total fat area | 1.08 | 0.88 | 1.33 | 0.47 |
| Muscle fat volume | 0.93 | 0.75 | 1.15 | 0.49 |
| Muscle area at L3 level | 0.93 | 0.73 | 1.17 | 0.52 |
| Subcutaneous fat area | 1.08 | 0.86 | 1.34 | 0.52 |
| Visceral fat volume | 0.95 | 0.77 | 1.16 | 0.61 |
| Visceral fat area | 1.05 | 0.85 | 1.30 | 0.64 |
| Total body volume | 0.97 | 0.78 | 1.20 | 0.76 |
| Total fat volume | 0.97 | 0.79 | 1.19 | 0.80 |
| Subcutaneous fat volume | 1.00 | 0.80 | 1.23 | 0.97 |

Supplementary Table S6. Univariable Cox analysis of clinical predictors of overall survival.

*HR* hazard ratio; *95% CI* 95% confidence interval*; CLIP score* Cancer of the Liver Italian Program score; *CPS* Child-Pugh Score; *AFP* α-fetoprotein as a binary variable (cut-off 400 ng/mL), *BCLC* Barcelona Clinic Liver Cancer stage.

| Variable | HR | 95% CI (Lower) | 95% CI (Upper) | *p* value |
| --- | --- | --- | --- | --- |
| CLIP score | 1.43 | 1.15 | 1.77 | <0.01 |
| Binary muscle density indicator (at L3 level) | 0.43 | 0.25 | 0.75 | <0.01 |
| TNM stage | 1.26 | 1.05 | 1.52 | 0.01 |
| Average 2D muscle density | 0.79 | 0.65 | 0.98 | 0.03 |
| CPS | 1.61 | 0.97 | 2.68 | 0.07 |
| Okuda stage | 1.38 | 0.89 | 2.15 | 0.15 |
| AFP | 1.35 | 0.84 | 2.18 | 0.21 |
| BCLC stage | 1.20 | 0.91 | 1.59 | 0.21 |
